# Cyclosporine A stimulated hair growth from mouse vibrissae follicles in an organ culture model

**DOI:** 10.7555/JBR.26.20110067

**Published:** 2012-03-29

**Authors:** Wenrong Xu, Weixin Fan, Kun Yao

**Affiliations:** a Department of Dermatology, the First Affiliated Hospital of Nanjing Medical University, Nanjing, Jiangsu 210029, China;; b Department of Microbiology and Immunology, Nanjing Medical University, Nanjing, Jiangsu 210029, China.

**Keywords:** cyclosporine A (CsA), hair follicle, mouse vibrissae

## Abstract

Hypertrichosis is one of the most common side effects of systemic cyclosporine A therapy. It has been previously shown that cyclosporine A induces anagen and inhibits catagen development in mice. In the present study, to explore the mechanisms of cyclosporine A, we investigated the effects of cyclosporine A on hair shaft elongation, hair follicle cell proliferation, apoptosis, and mRNA expression of selected growth factors using an organ culture model of mouse vibrissae. In this model, cyclosporine A stimulated hair growth of normal mouse vibrissae follicles by inhibiting catagen-like development and promoting matrix cell proliferation. In addition, cyclosporine A caused an increase in the expression of vascular endothelial growth factor (VEGF), hepatocyte growth factor (HGF), and nerve growth factor (NGF), and inhibited follistatin expression. Our findings provide an explanation for the clinically observed effects of cyclosporine A on hair growth. The mouse vibrissae organ culture offers an attractive model for identifying factors involved in the modulation of hair growth.

## INTRODUCTION

Cyclosporine A (CsA) is a hydrophobic cyclic undecapeptide produced by the fungus *Tolypocladium inflatum* Gams. It is a potent T cell-specific immunosuppressant, and is used successfully in various organ transplantations. In dermatology, CsA has been used successfully as the primary treatment for psoriasis[1]–[3], pyoderma gangrenosum, Behçet's disease[4], atopic dermatitis[5] and lichen planus[6]. Dose-dependent hypertrichosis is present in 80% of patients treated systemically with CsA. This side effect is usually noted approximately two months after the start of therapy. The underlying pathogenesis of this widely observed phenomenon is, however, unknown. Reports on the use of CsA for hair growth disorders sound conflicting[7],[8].

Cosmetically acceptable results were obtained in about 50% of the patients with alopecia areata, alopecia totalis, or alopecia universalis treated systemically with CsA (at a daily dose of 6 mg/kg)[9]. However, there are several reports of progressive alopecia areata following systemic CsA treatment[10]. The topical use of CsA for male pattern alopecia and alopecia areata has not been successful[11]. Besides the hair growth-promoting effects, stimulation of hair pigmentation has been reported in a 77-year-old patient[12].

In animal experiments, CsA delays hair shedding in human skin grafted onto nude mice[13], and prolongs human hair growth *in vitro*[14]. It has been shown previously that CsA stimulates telogen follicles to enter anagen in normal mice, when administered in high doses by topical or systemic administration[15], and inhibits dexamethasone-induced catagen development after intraperitoneal administration[16],[17]. Topically applied CsA protects rats from local alopecia induced by chemotherapy[18], but the underlying mechanisms of this effect are unknown. The purpose of the current study was to investigate the effects of CsA on hair shaft elongation, hair follicle keratinocyte proliferation, and mRNA expression of selected growth factors using an organ culture model of mouse vibrissae.

## MATERIALS AND METHODS

### Animals

Male and female C57BL/6 mice were raised under standardized conditions at the University Hospital Eppendorf or the First Affiliated Hospital of Nanjing Medical University. Mice aged 35-75 d were sacrificed and used for isolation of vibrissae follicles. The study protocol was approved by the local institutional review boards and animal experiments were carried out in accordance with the established institutional guidelines regarding animal use and care.

### Culture of vibrissae follicles

Mouse vibrissae follicles were isolated from the mystacial pad as described previously[19]. The mystacial pad was cut into three sides, and the skin flap was clamped and rolled backwards to expose the vibrissa follicles. Under a dissecting microscope, each follicle was fixed at the point where it penetrated the skin surface, and was removed from the surrounding tissue. The isolated follicles were placed immediately into Williams' medium E (Gibco Life Technologies, Hamburg, Germany). Those follicles with fine growing fibers were used and transected in their upper third.

The isolated mouse vibrissae follicles were randomized into groups with 30 follicles per group. The follicles were submerged individually in 1 mL Willams' medium E supplemented with 2.0 µmol/L glutamine, 100 U penicillin, 100 µg streptomycin, 2.5 µg fungizone (Gibco Life Technologies, Hamburg, Germany) and 10 ng sodium selenite (Sigma, Hamburg, Germany) in a 24-well plate.

A stock CsA solution with a concentration of 10^−3^ mol/L was prepared by dissolving 10 mg CsA in 666 µL of ethanol followed by addition of 160 µL of Tween 80 and 7,403 µL of the cold culture medium. The stock solution was diluted further in culture medium to yield final CsA concentrations of 10^−10^-10^−6^ mol/L. Those treated with vehicle only served as controls. Vibrissae follicles were cultured at 37°C and 5% CO_2_ for 10 d. The medium was changed every other d. For neutralizing antibody experiments, follicles were cultured together with CsA and anti-VEGF (0.2 g/mL, ab1316, Abcam Cambridge, MA, USA) or anti-HGFR antibodies (1.5 g/mL, ab10728, Abcam). The length of the hair shafts of each follicle was measured every other d. Follicles were harvested, which were then embedded in Tissue-Tek^®^ (SAKURA, Zoeterwoude, Netherlands) and stored at -80°C, or frozen in liquid nitrogen for total RNA extraction.

### 3 H-TdR incorporation assay

3H-TdR was added to each well and incubated further for another 16 h. Follicular cells were harvested, and each sample received 40 µL of glacial acetic acid, washed by 5% trichloroacetic acid (TCA) and then dehydrated by ethanol. Radioactivity (CPM, counts per min) was measured by scintillation counting (1219 RackBeta Liquid Scintillation Counter, LKB Instruments, Mt Waverley, VIC, Australia Lkb Wallac).

### Total RNA isolation and first-strand cDNA synthesis

RNeasy^®^ Mini Kit (Qiagen, Hamburg, Germany) was used to isolate and purify total RNA according to the manufacturer's protocol. cDNA was synthesized from total RNA by using First Strand cDNA Synthesis Kit for RT-PCR (AMV, Boehringer Mannheim, Hamburg, Germany). The synthesized products were stored at -80°C until PCR amplification.

### Polymerase chain reaction (PCR)

The specific target cDNA was amplified using the PCR Core Kit (Roche, Germany) and the following primers were used. Vascular endothelial growth factor (VEGF) (GenBank accession No. M95200): 5′-CAGGCTGCTGTAACGATGAA-3′ (sense), 5′'-AATGCTTTCTCCGCTCTGAA-3′ (antisense)[20]; hepatocyte growth factor (HGF) (Gen Bank accession No. D10213)[21]: 5′-CCATGAATTTGACCTCTATG-3 (sense), 5′-ACTGAGGAATGTCACAGACT-3′ (antisense); nerve growth factor (NGF) (GenBank accession No.V00836)[22]: 5′-CTAGTGAACATGCTGTGCC-3′ (sense), 5′'-CATGGACATTACGCTATGC-3′ (antisense); follistatin (GenBank accession No. X83377): 5′-TCCAACATCACCTGGAAGGG-3′ (sense), 5′-GCATTGTCACTGGCACAGAC-3′ (antisense). The PCR was performed in a Perkin Elmer 2,400 Thermal Cycler. The PCR products were electrophoresed and photographed under UV light with a digital camera (KODAK, DC120 ZOON).

As a control, RT-PCR amplification of β-actin was performed using the following two pairs of primer: sense: 5′-TCAGAAGGACTCCTATGTGG-3′ and antisense: 5′-TCTCTTTGATGTCACGCACG-3′, which yielded a 500-bp product, and sense: 5′-TGTTACCAACTGGGACGACA-3′ and antisense: 5′-TCTCAGCTGTGGTGGTGAAG-3′, which yielded a 392-bp product (GenBank accession No. M12481). Negative controls consisted of the reaction mixture without cDNA, or total RNA from lysates of vibrissa follicle.

### Ki67/terminal deoxynucleotidyl transferase (TdT)-mediated dUTP-biotin nick end labeling (TUNEL) double staining

A previously described double staining method was used[23]. Cryostat sections of 7 µm thick were fixed and incubated with digoxigenin-dUTP according to the manufacturer's protocol (ApopTag^®^ In Situ Apoptosis-Kit, Int. Ergen, Hamburg, Germany). This was followed by incubation with a rabbit polyclonal antibody against mouse ki67 antigen (Dianova Company, Hamburg, Germany). Subsequently, the TUNEL-positive cells were visualized by FITC-conjugated anti-digoxigenin F(ab)_2_ fragments, and the ki67-positive cells were detected with a TRITC-labeled goat-anti-rabbit antibody (Jackson ImmunoResearch Laboratories, Wext Grove, PA, USA). The nuclei were counterstained with DAPI[24]. Finally, sections were mounted using VectaShield mounting medium (Vector Laboratories, Burlingame, CA, USA). For positive controls, tissue sections from mouse spleen were used. Negative controls for TUNEL staining were made by omitting TdT. Sections were examined under a Zeiss Axioscope microscope (Jena, Germany) using the appropriate excitation-emission filter systems.

### Statistical analysis

The data were expressed as mean±SD and analyzed using version 11.0 of the SPSS software package (SPSS, Inc, Chicago, IL, USA). Statistical significance was estimated using Student's *t*-test, analysis of variance (ANOVA) and chi-square test with a *P*-value < 0.05 considered indicative of a statistically significant difference.

**Fig. 1:**
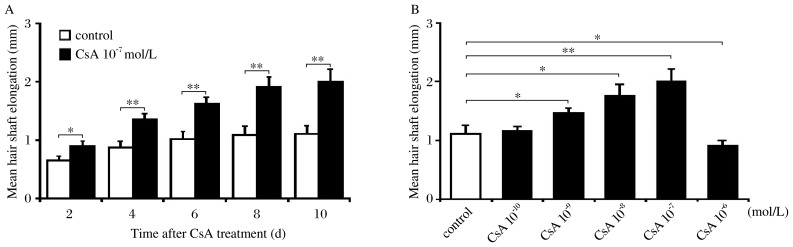
A: Time course of hair shaft elongation in isolated mouse vibrissae follicles treated with 10^−7^ mol/L cyclosporine A (CsA) or vehicle. B: Hair shaft elongation at d 10 as a indicator of the CsA concentration. The length of hair shaft is described as mean±SD; *P < 0.05, **P < 0.01.

## RESULTS

### CsA promotes hair shaft elongation and pre-vents development of catagen-like changes

Vibrissae follicles from 20 C57BL/6 mice were studied. Over 10 d of culture, hair shafts increased in length by approximately 0.21 mm/d in the group treated with 10^−7^ mol/L CsA, and by 0.11 mm/d in the control group treated with vehicle. The average elongations of hair shafts were 0.90, 1.36, 1.63, 1.92, and 2.01 mm in follicles cultured with 10^−7^ mol/L CsA, whereas 0.65, 0.88, 1.02, 1.09, and 1.11 mm in the control group on d 2-10. Statistically significant differences were observed (*P* < 0.05, ***Fig. 1A***).

Isolated mouse vibrissae follicles were cultured over a range of CsA concentraitons (10^−10^-10^−6^ mol/L). The average elongations of hair shaft were 1.16, 1.47, 1.76, 2.01 and 0.91 mm in the groups treated with CsA at concentrations of 10^−10^-10^−6^ mol/L on d 10, whereas 1.11 mm in the control group. At the lowest concentration (10^−10^ mol/L), CsA had no effect on hair shaft elongation, while higher concentrations of CsA stimulated hair shaft elongation. The highest concentration (10^−6^ mol/L) caused an inhibition of hair shaft elongation (***Fig. 1B***).

**Fig. 2:**
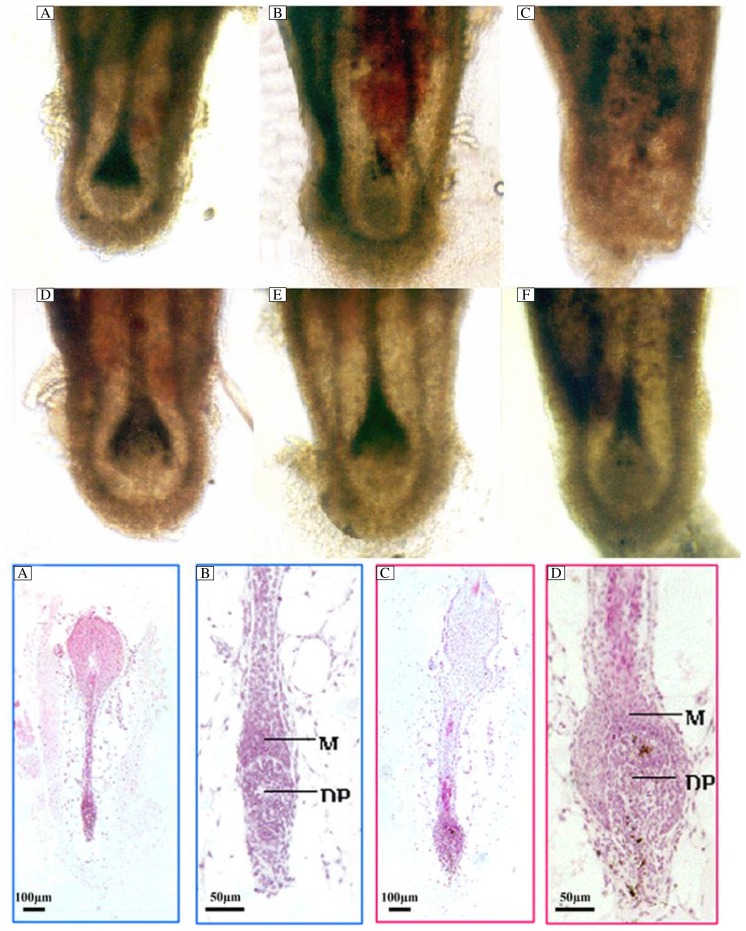
Bulbs of isolated mouse vibrissae follicles on d 2 (A, D), 6 (B, E) and 10 (C, F) of culture. Follicles are cultured with 10^−7^ mol/L cyclosporine A (D-F), or with vehicle (A-C). On d 10 of culture the whole shaft is either rejected from the follicle (C) or displays morphological signs in early catagen (F). Hematoxylin and eosin staining of isolated vibrissae follicles cultured for 7 d with 10^−7^ mol/L cyclosporine A (I, J) or vehicle (G, H). In most of vibrissae follicles treated with vehicle, the hair bulb becomes narrow and the dermal papilla is not surrounded by the epidermal matrix cells. The hair bulb looks like club hair. The dermal papilla has been compressed (G, H), while most of the vibrissae follicles treated with 10^−7^ mol/L cyclosporine A display full and round hair bulbs. The hair bulb is surrounded by epidermal matrix cells (I, J).

Microscopic analysis revealed morphologically unaltered hair bulbs on d 2 and 6 of culture. On d 10, however, the hair bulbs displayed morphological characteristics of follicles in early catagen. In some cases, rejected hair shafts were observed. After 10 d of culture, 63% (19/30) of vibrissae follicles showed rejection of hair shafts in the vehicle control group, while only 27% (8/30) were observed in the group treated with 10^−7^ mol/L CsA (*P* < 0.01). Half of the hair follicle bulbs displayed morphological characteristics in early catagen in CsA-treated group, whereas only 13% (4/30) in the vehicle control group (***Fig. 2A-F***).

**Table 1:** The results of ^3^H-TdR incorporation into the hair bulbs

CsA concentration	CPM±SD1
10^−6^ mol/L	17.44±2.50**
10^−7^ mol/L	150.17±12.37**
10^−8^mol/L	157.44±20.13**
10^−9^mol/L	120.50±14.01**
10^−10^mol/L	74.22±9.56**
Vehicle control	44.83±3.82**

Compared with the vehicle control, *P < 0.05, **P < 0.01. CPM: counts per min.

Cryostat sections of isolated vibrissae follicles were stained with hematoxylin and eosin on d 7 of culture. The hair bulbs of many follicles were thinner, and a few matrix cells covered completely the dermal papilla. The dermal papilla became smaller in anagen hair follicles, compared with the morphology of spontaneous developed catagen hair follicles. By contrast, many follicles treated with CsA had well-developed dermal papillae that were almost completely surrounded by matrix keratinocytes (***Fig. 2G-J***).

^3^H-TdR incorporation was used to test the correla-tion between the increase in hair shaft elongation and follicular cell proliferation. CsA over 10^−9^ to 10^−7^ mol/L significantly stimulated ^3^H-TdR incorporation into DNA in hair follicles (***Table 1***).

### Ki67/TUNEL double staining

**Fig. 3:**
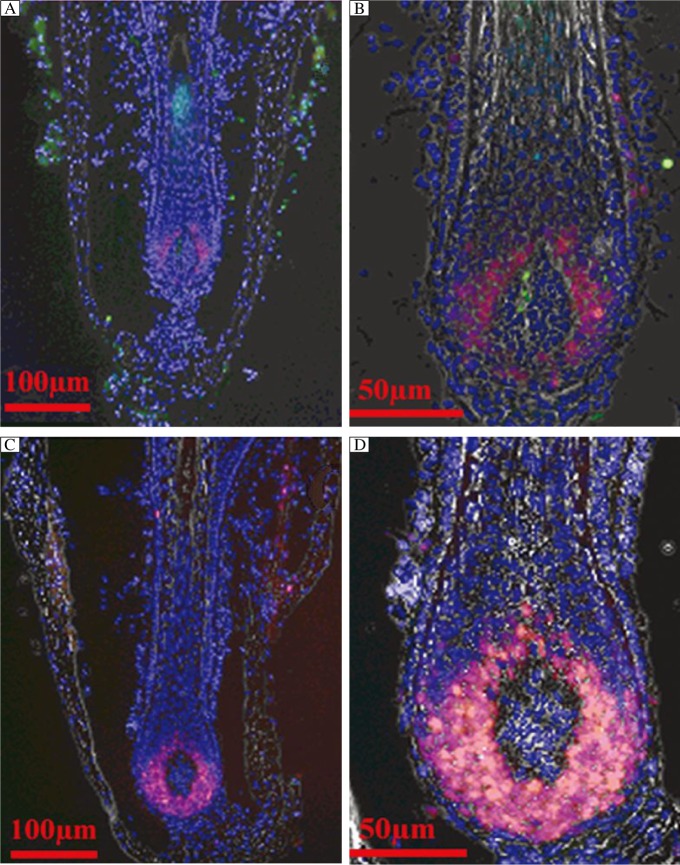
Ki67 and TUNEL double staining in cultured hair follicles treated with 10^−7^ mol/L cyclosporine or vehicle on d 5 of culture. Red staining shows ki67-positive cells. Green staining corresponds to TUNEL-positive cells. Cells are counterstained blue with DAPI. A, B: vehicle control; C, D: follicles treated with cyclosporine A.

Ki67/TUNEL double staining results were in accordance with the anagen or early catagen, and advanced catagen follicles (***Fig. 3***). On d 5, the average number of Ki67-positive cells was 180±9 cells per follicle in follicles treated with 10^−7^ mol/L CsA, which was significantly higher than that (78±14 cells per follicle) in the vehicle control (*P* < 0.05). Single TUNEL-positive cells were visible both in the hair matrix bulb and the outer root sheath, and in the dermal papillae. More TUNEL-positive cells (19±2 cells per follicle) were observed in the vehicle control group than those in the CsA-treated group (8±1 cells per follicle) (***Fig. 3***).

**Fig. 4:**
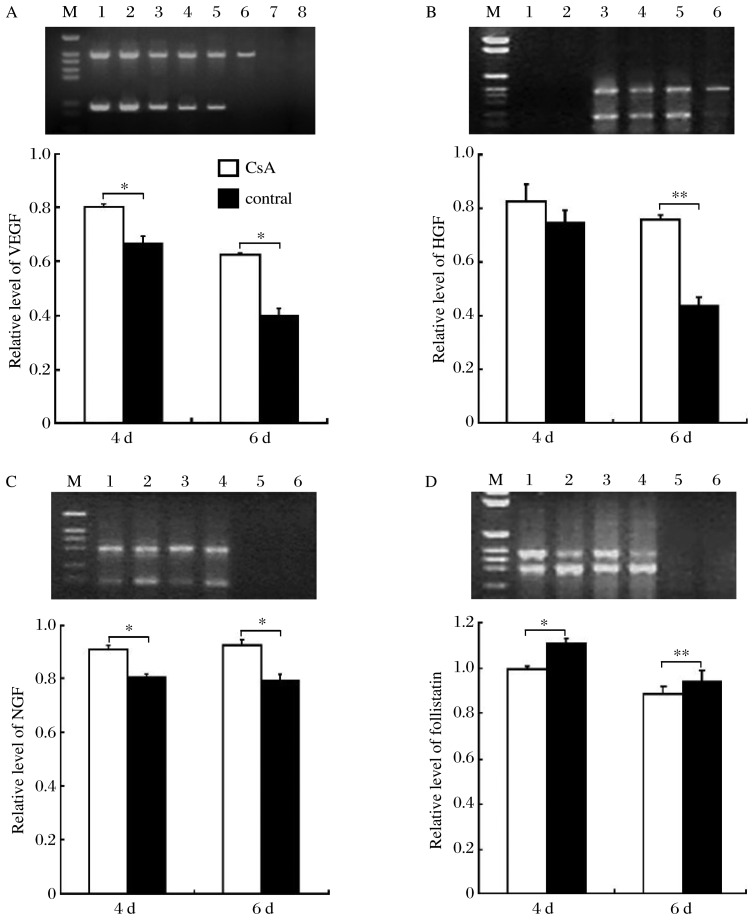
RT-PCR analysis of *VEGF, HGF, NGF* and *follistatin* mRNA expressions in cultured mouse vibrissae follicles. A Upper panel: RT-PCR for *VEGF* (207 bp) and *β-actin* (500 bp) in cyclosporine A (CsA)-treated group (Lane 1, 3, and 5), and the vehicle control group (Lane 2, 4, and 6) on d 2, 4 and 6, respectively. Lane 7 shows extracted mRNA, and Lane 8 shows the PCR product without cDNA templates. Lower panel: VEGF expression at d 4 and 6 post CsA treatment was normalized against beta-actin. B Upper panel: RT-PCR for *HGF* (263 bp) and *β-actin* (500 bp) in the CsA-treated group on d 2 (Lane 3) and 4 (Lane 4), and the vehicle control group on d 2 (Lane 5) and 4 (Lane 6). Lane 2 shows the extracted mRNA, and Lane 1 shows the PCR product without cDNA templates. Lower panel: HGF expression at d 4 and 6 post CsA treatment was normalized against beta-actin. C Upper panel: RT-PCR for *NGF* (216 bp) and *β-actin* (392 bp) in CsA-treated group (Lane 2 and 4), and the vehicle control group (Lane 1 and 3) on d 2 and 4, respectively. Lane 5 is extracted mRNA; lane 6 shows PCR product without cDNA templates. Lower panel: NGF expression at d 4 and 6 post CsA treatment was normalized against beta-actin. D Upper panel: RT-PCR for *follistatin* (500 bp) and *β-actin* (392 bp) in CsA-treated group (Lane 2 and 4), and the vehicle control group (Lane 1 and 3) on d 2 and 4, respectively. Lane 5 is extracted mRNA; lane 6 shows the PCR product without cDNA templates. Lower panel: follistatin expression at d 4 and 6 post CsA treatment was normalized against beta-actin. The mean density value for mRNA expression of *VEGF, HGF, NGF* and *follistatin* on d 4 and 6 of culture, using a Kodak digital camera and Kodak Digital Science 1DTM software. The expression levels are described as mean±SD; Compared with the control, *P < 0.05.

### CsA alters mRNA expression of *VEGF, HGF, NGF* and *follistatin*

On d 6 of culture, *VEGF* mRNA was expressed in the hair follicles treated with 10^−7^ mol/L CsA, while no expression was detected in the vehicle-treated follicles. A time-dependent increase of mRNA expression was found in hair follicles treated with 10^−7^ mol/L CsA between d 4 and 6 (*P* < 0.05, ***Fig. 4A***). On d 4 of culture, no *HGF* was detected in the vehicle control group, while significant amounts of *HGF* mRNA were expressed in the hair follicles treated with 10^−7^ mol/L CsA (*P* < 0.01) (***Fig. 4B***). Significantly higher expressions of *NGF* mRNA were observed in CsA-treated hair follicles on d 2 and 4 compared with the controls (*P* < 0.05) (***Fig. 4C***). On d 2 and 4 after incubation, the expressions of *follistatin* mRNA were significantly lower in the CsA-treated vibrissae follicles than those in the control follicles (*P* < 0.05 and *P* < 0.01, respectively) (***Fig. 4D***).

**Fig. 5:**
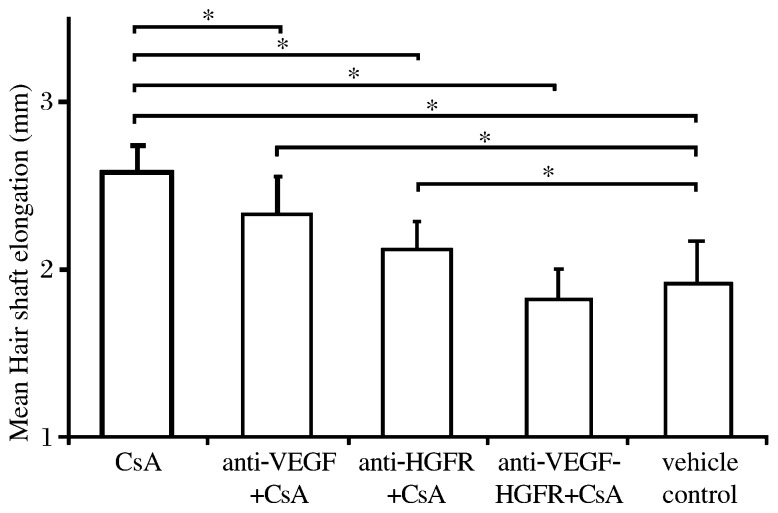
Vibrissae follicles cultured with CsA, anti-VEGF antibody, and anti-HGF-receptor antibody on d 4 of culture. The concentrations of these reagents are: CsA, 10^−7^ mol/L; anti-VEGF-antibody, 0.2 µg/mL; anti-HGFR-antibody, 1.5 µg/mL. M: DNA molecular weight marker.

### VEGF- and HGF receptors-neutralizing anti-bodies attenuate CsA-stimulated shaft elon-gation

When the isolated vibrissae follicles were cultured in CsA together with anti-VEGF (0.2 µg/mL) and anti-HGFR antibodies (1.5 µg/mL), the elongation hair shaft was inhibited significantly, and a significantly higher effect was achieved by using anti-HGFR antibody than by anti-VEGF antibody. In addition, a significant synergistic inhibitory effect was observed when both antibodies were used simultaneously (***Fig. 5***).

## DISCUSSION

CsA has shown hair growth-promoting effects in clinical and experimental applications[7]–[9]. Studies in a mouse model indicated that topical application of CsA induced anagen[25]. Here, we used a culture model of mouse vibrissae follicle to further elucidate the effect of CsA on hair growth. Our findings indicated that CsA over a concentration range of 10^−9^-10^−7^ mol/L stimulated significantly hair shaft elongation in the model, which is significantly different from the results of a previous study, indicating that CsA stimulated hair growth in nude mice[26]. This contradiction might be attributable to the different concentrations of CsA used. The results showed that CsA at concentrations of more than 10^−7^ mol/L inhibited hair growth, possibly due to the toxicity to hair follicle cells. Moreover, the effect of CsA on nude mouse vibrissae follicles is not comparable to that on normal hair follicles, since the former are highly abnormal in their structures[27],[28].

The growth of hair in humans is regulated by complicated mechanisms[29], including those factors that influence individual follicle cells, and exert effects on hair growth. The pathway of CsA-induced hair growth is not clearly understood. CsA inhibits the proliferation of normal and transformed keratinocytes[30], and therefore has beneficial clinical effects on psoriasis. However, the inhibitory effect of CsA on epidermal cell proliferation is contrary to its stimulatory effect on hair growth. In target cells (T cells), CsA binds to specific proteins (cyclophilin), and then is translocated into the nucleus and affects the transcription of interleukin-2 (IL-2). Besides the effect on IL-2 transcription, CsA also modulates the production of other cytokines like IL-1, IL-3, interferon-γ, granulocyte-macrophage colony-stimulating factor and tumor necrosis factor. IL-1 has recently been suggested as a crucial mediator inducing cessation of hair growth[31],[32]. The regulation of Ca^2+^- and K^+^-channels by CsA has also been suggested to be involved in hair growth stimulation[33].

Another possible mechanism for this action might be the direct and indirect effects of CsA on various growth factors. In the present study, we investigated the effects of CsA on mRNA expression of 4 key factors that influenced hair growth cycle[34]. Expressions of HGF and VEGF were stimulated significantly by CsA. The involvement of these two growth factors in CsA-induced hair shaft elongation was further supported by our observation that anti-VEGF and anti-HGFR antibodies inhibited the effect of CsA. VEGF was expressed in hair matrix and external root sheath, and in the dermal papillae. It is concluded that VEGF is synthesized by follicular cells and is stimulated by CsA. VEGF may be responsible for the growth and maintenance of perifollicular blood vessels[24], and affects the growth of dermal papilla fibroblasts *in vitro*[35]. NGF and its receptors have also been described to play an important role in hair follicle development and cycling. The present study showed that CsA significantly stimulated *NGF* mRNA expression in cultured mouse vibrissae follicles.

It is interesting to note that the expression of follistatin mRNA on d 4 of culture was significantly lower in follicles treated with 10^−7^ mol/L CsA. Follistatin belongs to the TGF-β superfamily, and at least three types of CsA-binding proteins are detectable in hair follicles[36]. One of these CsA-responsive genes is transcriptionally suppressed, which is consistent with our findings, indicating that CsA inhibited follistatin expression.

The current study also indicated that mouse vibrissae follicles in organ culture provide an attractive model for exploring the hair growth-modulating effects of CsA. It is thought that CsA stimulates hair growth in normal mouse vibrissae follicles by inhibiting a catagen-like development, and promoting matrix cell proliferation. This effect is possibly mediated, at least in part, by the enhancement of VEGF, HGF and NGF expression, and the inhibition of follistatin expression.
